# ‘Everybody’s voice is important’: using translational simulation as a component of change management

**DOI:** 10.1186/s41077-025-00364-0

**Published:** 2025-07-05

**Authors:** Nathan Oliver, Kathryn Twentyman, Katie Howie

**Affiliations:** 1 University of Canberra, Canberra, Australia; 2 NHS Lothian, Edinburgh, United Kingdom

**Keywords:** Translational simulation, Change management, Systems testing

## Abstract

**Background:**

Changes in healthcare systems are often highly stressful experiences for healthcare teams, contributing to disengagement and resistance to change. Translational simulation has been shown to be impactful at both organisational and department-based levels; however, its impact on the experience of change for frontline staff has not, to date, been explicitly explored. Understanding the impact of translational simulation on the perception of teams exposed to healthcare system changes, and how to optimise our approaches to support change management on a team and individual level, may be the difference between an overwhelmed and disengaged workforce and a positive and engaged one.

**Methods:**

We used template analysis as an analytic tool to gain new understanding of the impact of translational simulation on the experiences of staff members undergoing change. Utilising Bartunek et al.’s (2006) conceptual framework to inform the priori themes of our template, we interviewed nine Registered Nurses involved in a major relocation into a purpose-built paediatric hospital in Edinburgh, UK. We sequenced the interviews to take place in the lead up to a planned simulation event, with a follow up second interview 1 month after the hospital move. On the day of the simulation, we additionally collected a series of ‘headline’ thoughts from the group across the simulation to track their thoughts and feelings toward the move. Interviews and ‘headlines’ were recorded, transcribed, and thematically analysed using template analysis methods.

**Results:**

Our findings demonstrate that the use of translational simulation significantly enhanced staff preparedness and engagement during a major hospital relocation, suggesting that incorporating such approaches can be a valuable component of change management strategies in healthcare settings.

**Conclusions:**

Whilst further research is required, these findings promote the considered use of translational simulation as a potentially significant component of the change management process.

**Supplementary Information:**

The online version contains supplementary material available at 10.1186/s41077-025-00364-0.

## Introduction

Organisational change within the healthcare systems is often highly stressful for healthcare teams and can contribute to disengagement and even a sense of resistance [1–4]. Translational simulation has been shown to be impactful at both organisational and department-based levels; however, its impact on the experience of change for frontline staff has not been explicitly explored [5–10]. Understanding how translational simulation can support teams during times of organisational change—and being intentional about its use—may make the difference between a team that feels stressed and disengaged and one that feels supported and motivated.

**Fig. 1:**
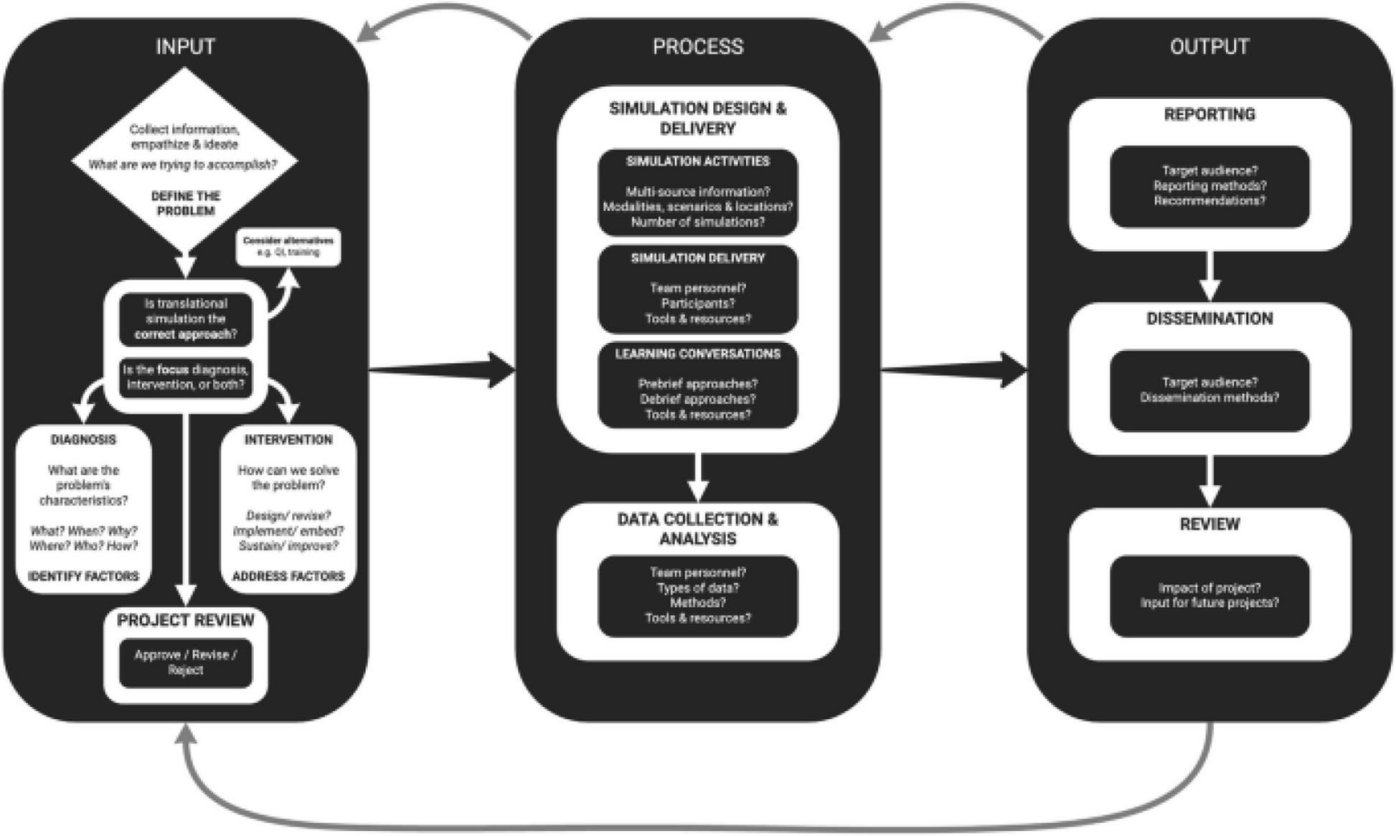
Input-process-output framework for translational simulation (licensed under a Creative Commons Attribution 4.0 International License https://creativecommons.org/licenses/by/4.0/) (5)

Translational simulation is a methodological approach aimed at utilising simulation as a tool to inductively interrogate systems to optimise processes or mitigate latent safety threats [8, 10]. Nickson et al. describe a three-phased process of working with key stakeholders to (i) ensure an in-depth understanding of the requirements of a system in review (input), (ii) develop a robust simulation intervention along the agreed testing parameters (process), and (iii) strategically report, disseminate, and review important findings which can then be translated into practice change and optimisation (output) [5, 11]. An illustration of the input-process-output framework can be seen below (see Fig. 1). Practical examples of using such an approach have been seen in the testing of both clinical spaces during the COVID pandemic and new clinical bundles prior to clinical roll out [6, 10–12]. These papers demonstrate the impact that translational simulation can have and the role it should play in macro and meso levels of planning and implementing change within the healthcare setting. What these pieces have not yet explored is the role translational simulation may have more specifically on the change experience of individuals and their teams.

Change management practices are well established within the literature, alongside a range of theoretical models to support industries in mitigating risks when change is required [13, 14]. The simulation literature is no stranger of considering change management theory in the context of healthcare simulation—with several important pieces published in recent years [10, 15, 16]. One criticism of such models, however, is that there is often a greater focus on the leadership of change at organisational (macro), and department (meso) levels, and less so to the experience of individuals (at a micro level) who have historically been categorised according to their levels of resistance [17]. Ford et al. (2008) challenge the reader here to reconsider ‘resistance’ from an irrational and dysfunctional set of emotions into a relational opportunity to promote sensemaking across the parties [3]. Taking this further, Choi et al. (2022) sought to understand what resources might be of value in seeking to improve individual perceptions of readiness in the context of change in a healthcare setting. The authors found whilst various top-down approaches (from regular updates and informational resources) were considered important, a simple phone call check-in, opportunities for group discussion, and appropriate time-and-space amidst busy workloads were equally essential during times of organisational change [18]. A sense of intentionality in offering affordances and opportunities to allow individuals to prepare for change is key [17–19]. Nilsen et al. (2020) expounds on the importance of *involving* healthcare teams into change processes, and uncertainty and increased stress related leave and disengagement when the opportunity to engage in sensemaking activities is not appropriately considered [1].

**Fig. 2:**
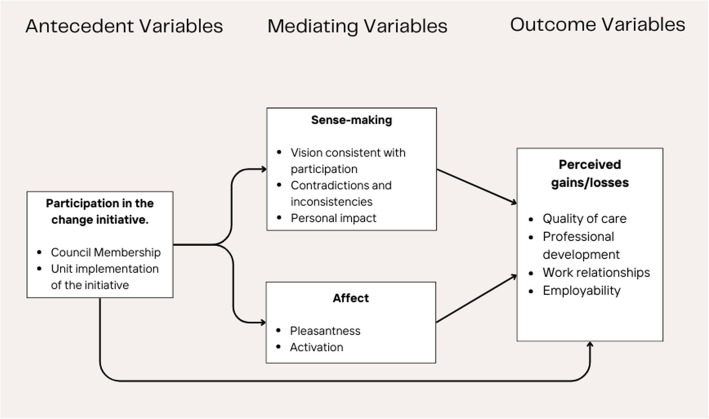
Bartunek et al. (2006) Conceptual Framework [17] (Reproduced with permission from Sage Publications)

Bartunek et al. (2006) explicitly focus on the individual’s experience of organisational change and advocate thoughtful ‘shared governance’ as a key feature of supporting individuals and teams [17]. By ‘shared governance’, the authors refer to the process of including and involving those impacted in the change process itself. They propose a conceptual model (see Fig. 2) which articulates a set of variables that influence the way change might be experienced at the individual level [17]:

1. Antecedent variables: these refer to the opportunities (which might take a variety of forms) for individuals to be engaged and involved in the change process at any given time [1, 17].
2. Mediating variables: these refer to the emotions and meanings that may emerge, shift, and change dynamically along the change process. A sense of clarity of vision, sensemaking of messaging, and perceptions of contradiction and mixed messages are all agents that impact experiences within this domain [17].
3. Outcome variables: these refer to the feelings associated with the change and the perceptions of its net gain or loss for the individual or team [1, 17].

Our study sought to examine how the implementation of a translational simulation programme impacted the *change experience* of a group of healthcare professionals during a hospital relocation; thus, we selected Bartunek’s model to explore this. Our research question was:

–In what way can translational simulation influence the process of sensemaking and adaptation to change, in the context of a hospital relocation?

## Methods

### Research design

Taking an interpretivist stance to our enquiry and noting the many multiple interpretations of individuals experiencing large-scale change (and indeed those researching it), we took a *contextual constructionist* position [20]. This position asserts that knowledge is locally and situationally dependant and ‘permeable’ to change based on experiences and encounters (Madill et al. 2000). In the same way as those participating in change will interpret the experience uniquely, so too will the research team as we collected, interpreted, and here present our data. Context matters. Therefore, our goal is to present the stories and experiences across the whole simulation intervention, and to present the findings as much as possible from the lips of research participants.

### Researcher characteristics and reflexivity

We developed a consistently reflexive approach across the research incorporating personal, interpersonal, methodological and contextual domains [21]. We brought a range of different experiences to the research table. NO has a nursing background, and KT and KH are both medically qualified. KH was a junior doctor working clinically within paediatrics, and therefore was directly impacted by the hospital relocation and was able to bring this lens to the research. All authors worked within NHS Lothian during the period of data collection and shared in many of the broader communities’ hopes and concerns about the impending move. NO was a regional leader of the simulation team within the Health Board and was able to offer experience and expertise in translational simulation; indeed, he was active in many of the simulation events and debriefs described within this paper. KT and KH were nominated to facilitate all facets of data collection, including interviews. Whilst our experiences in simulation and our context in living and working in the region added important value to our approaches, we reflected considerably across the research about our relative newness to implementation science and change management scholarship. This required us to deep dive into the literature and regularly reflect on our methodological decisions and was one driver to respond to the research question through a deductive approach.

### Context

In 2021, the paediatric hospital in Edinburgh moved from a traditional Victorian era hospital footprint, and to a new, ‘state of the art’ hospital site within a dedicated healthcare precinct. In preparation for this move, the local simulation team were invited to deliver a bespoke translational simulation project across the various departments and wards in support of the safe transition between sites, influenced by the input-process-output approach as outlined by Nickson et al. [5].

#### Input

From the outset of the project, the simulation team elected a public *mantra* that ‘everybody’s voice is important’. To this end, a series of paper-based questionnaires, as well as numerous informal in-person visits to frontline worker’s spaces (including nurses’ stations, reception and staff management offices, and doctors’ rooms) hospital-wide, were made over a series of weeks—seeking to better understand the key staff concerns surrounding the realities of relocation. Questionnaire data was analysed by the simulation team, and in keeping with Nickon et al.’s recommendations, a range of themed concerns was established in which translational simulation was well positioned to address both diagnostic (e.g. how families might most efficiently navigate an outpatient department*)* and interventional (e.g. location for emergency trolleys within the paediatric intensive care unit) issues (5). A series of scenarios was then designed to reproduce an ‘average day’ for each department within the new hospital.

#### Process

In keeping with valuing ‘everybody’s voice’, staff representing all available disciplines and levels were invited to participate in the ensuing six simulation half-days across various departments. These included admin and security staff, alongside the multidisciplinary clinical team, from the more junior through to senior leaders and management. Importantly, members of the community, parents, and children who utilised the services of the hospital were invited to attend and collaborate. Half-days included an orientation and tour of the environment, followed by a series of scenarios. Sessions were led by expert members of the simulation team, who chose a modified Scottish Centre Debriefing model to facilitate systems-focused team debriefs following each scenario [22].

#### Output

A detailed report was compiled after each event and distributed to department managers for dissemination. An executive summary report was additionally produced for the hospital executive leading the change. Reports consisted of rich summaries of input data eliciting staff concerns and considerations, scenario summaries, debrief summaries, and take-home-messages. Importantly, each report offered a list of latent safety threats which staff discovered as part of the simulation and debriefing phase. The importance of these reports, and the way they were strategically disseminated was key to the project and in keeping with evidence-based recommendations [5].

### Sampling strategy

Despite the broad participative approach of the translational simulation programme, as well our own epistemological value on a wide exposition of context, resource limitations required us to make some pragmatic decisions regarding the sample group of this study. We selected a purposeful sample of Registered Nurses as the participants of this study. Nurses within our context constituted the key substantive staff who could offer experiences both before and after the hospital relocation in a way that could meaningfully respond to the research question. We recruited twelve [12] nursing participants from a range of different medical and surgical departments across the hospital.

Inclusion criteria were the following:

1. A Registered Nurse with the Nursing and Midwifery Council
2. Substantively employed (i.e. on a long-term permanent contract, within the Health Service).
3. Clinically working throughout the transition period between hospital sites (i.e. having worked at both the old and the new hospital sites during the changeover period).

### Data collection methods

We utilised a three-phased approach in order to respond to the research question [20]. KT and KH facilitated the phases of data collection across the project. Phase one consisted of a series of one-on-one, semi-structured interviews, taking place within the workplace in the weeks leading up to the translational simulation intervention. Interviews were audio recorded, transcribed, and securely stored. Phase Two occurred throughout the day of the event itself, where we captured the dynamic ‘stream of thoughts’ of each participant to capture a ‘snapshot’ of their perceptions as the intervention progressed. We used Kolbe et al.’s (2018) ‘What’s the Headline in your Mind Right Now?’ approach, requesting that participants jotted Post-It™ Note reflections at several time points (specifically, after a tour of the relevant department, immediately following a translational simulation scenario, and following the structured debrief) [23]. Post-It™ Notes were transcribed verbatim for analysis and originals destroyed. Finally, we invited all 12 participants for a second interview 1 month after the hospital move.

### Data analysis

We used template analysis (TA) to inform our analysis, an appropriate method when utilising the contextual constructionist lens [24, 25]. Bartunek’s framework framed both our questions, as well as our priori themes (see Table 1). The deductive analysis was completed by the research team. The process of coding was consistent with the guidance from Braun and Clarke (2006) with TA-specific guidance from Symon et al. (2012) [25, 26].

**Table 1:** Priori themes applied to participant data, informed by Bartunek et al. [17]

Priori theme	Description
Antecedent variable	Includes: participation in change up to the translational simulation event
Mediating variable	Includes: notions of sense-making and affect in the lead up, during, or in reflection of the simulation event
Outcome variable	Includes: perceived gains or losses after the change as it relates to the simulation event

### Ethical considerations

In accordance with the Declaration of Helsinki, all participants were given detailed information of the study alongside a one-on-one discussion. Informed consent was obtained by all participants. Ethical approval was sought and waived by NHS Lothian Caldicott guardians.

## Results/findings

All 12 participants undertook an initial pre-interview and ‘Headline’ captures on the day, however only 9 participants were able to complete the post-interview. Participants not able to participate in this third phase were excluded from the study. Pre- and post-interview durations were between 9 and 30 min. In keeping with the values of a contextual constructionist approach, we present these in the original voice of participants as often as possible, shared through anonymous participant codes to identify representative quotations (e.g. P04) [20] (Table 2).

**Table 2:** Final template of themes, informed by Bartunek et al.[17]

1. Antecedent variable a. Participation in the change initiative b. Preparing for the simulation event 2. Mediating variable a. Sensemaking i. Being on the new site in person ii. Together as a team b. Affect i. Simulation to explore local logistics ii. Effective team debriefing 3. Outcome variable a. Quality of care b. Team relationships	

### Antecedent variables

#### Participation in the change initiative

[it’s] fear of the unknown. If we were kept up to date a bit more and knew what was happening, then we probably wouldn't feel as bad as we do—P01.

I feel like it’s so close, but we’ve not really been told anything—P01.

Within the pre-intervention interview phase, interview respondents expressed feelings of frustration and anxiety in the lead up to the imminent hospital relocation. Many situated these emotions around a perceived lack of consultation and involvement within the process with words such as ‘lost’ and ‘unsure’ noted as regular words used to describe their thoughts and feelings.

I don’t feel I’ve been very involved in it to be honest. The more senior people in the ward have but not, I don’t feel very involved no—P08.

I think it’s mainly we’re not really kept up to date on things like communication’s not the best and we sort of are the last to know. We still don’t know if we’re actually moving or not. Erm obviously last time it was like forty-eight hours before they’ve pulled the plug. So, we get to a stage where we’re packed up ready to go and then it comes out in the press before they tell us so, erm, yeah communications not been the best

… We’re supposed to know we’re moving in less than two weeks and yet we still feel as if it’s still a little bit vague and is it going to happen?—P09.

Not all responses were negative, with one of the participants for example speaking of how they *‘*went across a few weeks ago and as a whole team and they were really goodat like listening to everyone's ideas’ (P10), which was helpful in planning the move and transition in working.

I’m thinking we’re all new to this, everyone’s in the same boat which is actually quite nice. I don’t feel like a newbie or I’m not as qualified as them so may not be able to do this. It’s none of that, it’s just quite…yeah, I’m just quite excited for it, [figuring] things out—P05.

… Everybody’s voice is important just because you’re the senior manager doesn’t mean you’ve got all the good ideas, you know, just because you’ve got a PhD doesn’t mean you’ve got a jot of common sense about our processes—P06.

#### Preparing for the simulation event

One participant reflected that she was hopeful that the upcoming simulation event offered an opportunity to discuss and collaborate as a team; ‘we can all bounce off each other and hopefully answer some of our concerns and worries. And I thinkit’llmake us feel more confident about how things will go’ (P03).

Participants reflected the idea of the simulation event as a potential ‘levelling factor’, a reminder that the relocation impacted all team members regardless of discipline or professional level.

Despite the clearly prevalent feelings of anxiety prior to the move, participants felt reassured at the prospect of attending a simulation event as an opportunity to ‘[get] a sense of the ward and how it runs and how actually where we’re going to do things and I think that will be take less pressure off us’, and that ‘if things go wrong at least we can try to trouble shoot them in a safer kind of situation’ (P03).

### Mediating variables

#### Sensemaking

I have massive anxiety about is the change in our environment from what we have, where we can see the patients, we’ve got them all in full view, we can see the staff, the way the place runs. The new unit, we have essentially got four different areas to staff—P06.

Whereas pre-simulation interviews elicited elevated levels of stress and some frustration on the perceived communicative contradictions and levels of change between their old and new clinical environments, there was a noted shift in narrative within the simulation event. The team reported significant resonance in taking time within the simulation itself and, following debrief, to discuss novel staffing set-ups and approaches to care.

Actually, being here for the sim day gave all members of the team, an open, you know…, they had a time to see what it would be like, and I think that certainly worked wonders … and even after the sim day we had discussions around it.—P02.

… it let us think, oh that’s not going to quite work, and we actually need more staff on a night shift now.—P05.

Participants commented on feeling reassured through noticing, experiencing, and discussing concerns together within the simulated environment and the context this allowed for them to explore these, ‘in full view’, prior to the move.

#### Affect

I think there was a lot of anxiety about [the change in nursing style] but … being in the environment and seeing how it worked relieved a lot of that—P02.

There was consistent concern from participants during initial hospital tours in how new spaces might impact safe practice and staff well-being in the longer term (for example, the quantity and quality of dedicated staff areas in the hospital, including break spaces, changing rooms, and lockers). This was observed to be a significant area of stress.

Parking will be a challenge, … and not having a fridge on the ward. I know it seems ridiculous but it’s the little things—P06.

During and following the translational simulation, a noted change in responses was observed, with participants commenting via Post-It™ note:

- ‘[the simulation represents an] excellent opportunity to freely explore facilities’.
- ‘[I am feeling] good after [the] sim due to talking about logistics and working as part of a team to problem solve’.
- ‘[The opportunity to] troubleshoot is giving us more action plans and something we can work towards’.

Overwhelmingly, many participants’ affective responses described feeling ‘overwhelmed’, ‘apprehensive’, and ‘nervous’ during the initial department tours and both during and immediately after the simulation itself. However, after the structured debrief, participant responses appear to have reflected more positive responses, such as ‘[I am] excited to move, but still nervous’, (P08) and ‘[I was] feeling less anxious after discussion at end’ (P05).

One participant reflected that the simulation increased her anxiety ‘in some ways’ by making her realise the scale and number of changes and by prompting her to ‘consider about things [she] hadn’t thought of’ but that the debrief then assuaged some of these worries through open discussion; ‘I feel good knowing everyone is in the same position, and we will support each other in our team. The simulation was definitely a positive experience’—(P05).

### Outcome variables

Findings within this section overwhelmingly reflect data from interviews taken 1 month after hospital relocation.

#### Quality of care

Within post-interviews, participants reported that engaging in the simulation session offered an important preparatory feature for both them and their team, perceiving that the translational simulation afforded them conversations and decisions that optimised safety and the quality of care they felt subsequently able to provide.

Several reported finding it significantly easier to feel settled having ‘already worked through some of the practicalities of a normal working day’ (P03). This ranged from recognising the different alarm noises to learning how to contact someone, one participant reflecting ‘just the simple things I think made a difference’ (P05).

After the sim we had, it was in our group chat, our work group chat, which was really good … senior management had popped it in that things we had come across that maybe weren’t going to quite work like it did in the old hospital and things were maybe had to change—P05.

An unexpected finding from interviewee’s was the perception that those who participated in the simulation became *local experts* in the first few weeks after the site relocation – the local ‘*champions’* of quality change.

It got people involved in other things that might have happened [in different areas of the simulation and] there’s always been one person that was at the sim training who has said ‘actually we came across they during sim training’ and they’ll know the answer—P02.

One participant reported that staffing numbers had changed as a direct impact of the simulation activity, enhancing quality care from the first day of opening, noting that ‘it let us think, oh that’s not going to quite work, and we actually need more staff on a night shift now’ (P05).

We were all kind of bouncing off each other—P03.

#### Team relationships

Going back to the sim day, even without scenarios and things, I think having the chance to, as a team, go round and explore for themselves. You know one person might have found there is something in a cupboard that someone else never knew about. As a team (now), when you come together there’s always one person on shift that hascome across this before—P02.

Participants reflected that participation in the simulation created an opportunity to troubleshoot as a team. They reported a noted change within their work relationships, both during the simulation, and importantly remaining into the early days of their relocation.

Another participant reflected ‘I think we would have had a lot more questions if we hadn’t had the sim day. Yeah, cause people who hadn’t attended have asked but…between the team there’s always been someone or two people that have been on shift that were part of the sim day and have been able to reflect on that and say, what was good and what worked’ (P02).

Another participant’s reflection, which we as a research team found profound, was the observed emphasis on the event debrief: ‘it was a big group but actually everybody felt they could speak’ (P06). They went on elaborate that for them, this meant that the staff themselves felt empowered with the knowledge of how things were ‘meant to run’ and encouraged to take proactive steps to ensure the move went smoothly.

As a team, everyone was saying ‘you know we need to make sure this is working’—P02.

## Discussion

The organisational change reported within our study was a profound experience for all respondents—not only in the context of new work environments, new team dynamics, and in new ways of working, but on a very personal level. Participating in a series of translational simulations impacted their perception in the lead up to the change, impacted their perception on the day itself, and appears to have paid some important dividends a month after the change itself. Bartunek et al. emphasise the importance of participation and shared governance in any major change initiative, and how ongoing sense-making and emotions that are generated throughout profoundly impact the perception toward a positive and constructive approach to change [17]. Our findings illustrate this phenomenon and provide some important implications in using translational simulation as a potentially significant component of the change management process.

Despite regular hospital-wide updates and adherence to the appropriate change management process, participants in our study expressed a consistent perception of limited control over the impending move and a lack of engagement within the process. By participating in translational simulation, individuals were afforded the opportunity to engage with the change in a personal, and to them, a valuable way. At the organisational (macro and meso) level, individuals were able to collaborate with the change through their interrogation and mitigation of many implicit latent safety threats within their departments. From an individual level (micro), the opportunity to simulate their work and reflect as a team appears to have a strongly positive and ongoing impact on their sense of involvement and ability to have a say in some important and functional aspects of their department.

On reflecting upon the individual components of the simulation design process, we struggled to isolate any that had a greater influence on the results. Thoroughly and widely interviewing hospital members regarding their priorities and developing a ‘day in the life’ kind of simulation (aligning with the *input* stage from Nickson et al.’s work), allowed individuals to express their concerns and participate in what ‘mattered most to them’ [5]. The ward tour, followed by the systems-based simulation event, (aligning with the *process* stage from Nickson et al.’s work) generated opportunities of staff members to make sense of the change and even problem solve some the personal and environmental impacts these would have within their clinical teams [5]. A key feature that was noted to decrease a sense of anxiety in participants, was a structured debrief at the end of each scenario. This echoes what many have stated within the simulation literature regarding the importance of the debrief in any simulated activity [27–30].

A surprising finding for us was the perceived impact that translational simulation had a month after the organisational change. Whilst we might have expected such positive responses immediately after the simulation in isolation, the resonant experiences reported that came out of the event, even weeks after the move itself, appears to demonstrate two crucial factors.

- Firstly, it supports Bartunek’s conceptual model around the individual experience of change, and of considering the antecedent and mediating variables of individuals and teams when contemplating large scale change [17].
- Secondly, how translational simulation might be considered within the change leader’s toolkit as an impactful approach to influencing perceptions of change.

Our findings illustrate another role that translational simulation can play in healthcare as a potentially significant tool for change leaders. Where other change management communication processes often require a ‘top down’ approach—led by senior management with a trickledown effect to individuals—the use of translational simulation affords an opportunity for staff members themselves to be more explicitly involved in the change areas which impact them the most and in this sense empowered them to take proactive steps and make decisions, as well as ‘communicating up’ areas of risk to patients and staff.

### Limitations

We consider several limitations to our research. As we stated within the sampling strategy section, we acknowledge the potential contradiction in the simulation team’s mantra that ‘everybody’s voice is important’, and our pragmatic decision to focus on those of the nurses in the study itself. Future research surrounding other members of the multidisciplinary team, indeed with a full team reflecting together, would add to the evidence-base. We also acknowledge the wealth of change management and implementation science literature that exists, and the range of conceptual frameworks that might have added different and equally valuable perspectives to the one we selected. Future research incorporating different models and including experts within these domains within the authorship team would add significant value.

## Conclusion

Our study sought to examine how the implementation of a translational simulation programme impacted the *change experience* of a group of healthcare professionals during a hospital relocation. Our findings demonstrate that a thoughtfully designed programme, written inclusively and collaboratively with the whole healthcare team, can be impactful in the experience and perceptions of change. Understanding the impact of translational simulation on teams exposed to healthcare system changes, and how to optimise our approaches to support change management on a team and individual level, may be the difference between an overwhelmed and disengaged workforce and a positive and engaged one.

## Supplementary Information

Supplementary Material 1

## Data Availability

The data that support the findings of this study are not openly available due to reasons of sensitivity and are available from the corresponding author upon reasonable request.

## References

[1] P Nilsen I Seing C Ericsson SA Birken K Schildmeijer 2020 Characteristics of successful changes in health care organizations: an interview study with physicians, registered nurses and assistant nurses BMC Health Serv Res 20 1 147 32106847 10.1186/s12913-020-4999-8 PMC7045403

[2] JP Meyer TD Hecht H Gill L Toplonytsky 2010 Person–organization (culture) fit and employee commitment under conditions of organizational change: a longitudinal study J Vocat Behav 76 3 458 473

[3] JD Ford LW Ford A D'Amelio 2008 Resistance to change: the rest of the story Acad Manag Rev 33 2 362 377

[4] F Milella EA Minelli F Strozzi D Croce 2021 Change and Innovation in Healthcare: Findings from Literature Clinicoecon Outcomes Res 13 395 408 34040399 10.2147/CEOR.S301169 PMC8141398

[5] CP Nickson A Petrosoniak S Barwick V Brazil 2021 Translational simulation: from description to action Adv Simul (Lond) 6 1 6 33663603 10.1186/s41077-021-00160-6 PMC7930894

[6] V Brazil B Lowe L Ryan R Bourke C Scott S Myers 2020 Translational simulation for rapid transformation of health services, using the example of the COVID-19 pandemic preparation Adv Simul (Lond) 5 9 32514386 10.1186/s41077-020-00127-z PMC7267758

[7] Colman N, Doughty C, Arnold J, Stone K, Reid J, Dalpiaz A, et al. Simulation-based clinical systems testing for healthcare spaces: from intake through implementation. Advances in Simulation. 2019;4(1). 10.1186/s41077-019-0108-7 PMC6676572 31388455

[8] V Brazil 2017 Translational simulation: not'where?'but'why?'A functional view of in situ simulation Adv Simul (Lond) 2 1 20 29450021 10.1186/s41077-017-0052-3 PMC5806247

[9] V Brazil G Reedy 2024 Translational simulation revisited: an evolving conceptual model for the contribution of simulation to healthcare quality and safety Adv Simul (Lond) 9 1 16 38720396 10.1186/s41077-024-00291-6 PMC11080180

[10] Dubé M, Posner G, Stone K, White M, Kaba A, Bajaj K, et al. Building impactful systems-focused simulations: integrating change and project management frameworks into the pre-work phase. Advances in Simulation. 2021;6(1). 10.1186/s41077-021-00169-x PMC8082890 33926582

[11] N Colman JW Newman A Nishisaki M Register SE Gillespie KB Hebbar 2021 Translational simulation improves compliance with the NEAR4KIDS airway safety bundle in a single-center PICU Pediatr Qual Saf 6 3 e409 34046538 10.1097/pq9.0000000000000409 PMC8143778

[12] HMS Lababidi U Alzoraigi AA Almarshed W AlHarbi M AlAmar AA Arab 2021 Simulation-based training programme and preparedness testing for COVID-19 using system integration methodology BMJ Simul Technol Enhanc Learn 7 3 126 133 37534698 10.1136/bmjstel-2020-000626 PMC7316112

[13] Kotter JP. Leading Change: Why Transformation Efforts Fail. (cover story). Harvard Business Review. 1995;73(2):59–67.

[14] K Lewin 1947 Frontiers in group dynamics: concept, method and reality in social science; social equilibria and social change Human Relations 1 1 5 41

[15] Eller S, Rudolph J, Barwick S, Janssens S, Bajaj K. Leading change in practice: how “longitudinal prebriefing” nurtures and sustains in situ simulation programs. Advances in Simulation. 2023;8(1). 10.1186/s41077-023-00243-6 PMC9862849 36681827

[16] M Ortega Vega A Bignell K Virk OP O'Sullivan G Billon G Evans 2022 Developing simulated patients for online simulation: reflections on actor management and scenario adaptation Clin Simul Nurs 66 44 49

[17] Bartunek JM, Rousseau DM, Rudolph JW, DePalma JA. On the receiving end. J Appl Behav Sci. 2006;42(2):182–206.

[18] Choi KA, Lindert L, Schlomann L, Pfaff H."I'll leave that to the case managers."Healthcare service providers'perceptions of organizational readiness for change in a randomized controlled trial-a qualitative analysis exploring implementation success. Int J Environ Res Public Health. 2022;19(9). 10.3390/ijerph19095782 PMC9104361 35565177

[19] C Pomare K Churruca JC Long LA Ellis J Braithwaite 2019 Organisational change in hospitals: a qualitative case-study of staff perspectives BMC Health Serv Res 19 1 840 31727067 10.1186/s12913-019-4704-y PMC6857127

[20] A Madill A Jordan C Shirley 2000 Objectivity and reliability in qualitative analysis: realist, contextualist and radical constructionist epistemologies Br J Psychol 91 1 1 20 10717768 10.1348/000712600161646

[21] Olmos-Vega FM, Stalmeijer RE, Varpio L, Kahlke R. A practical guide to reflexivity in qualitative research: AMEE Guide No. 149. Med Teach. 2022;45(3):1–11. 10.1080/0142159X.2022.2057287 35389310

[22] Oliver N, Maran N, Edgar S, Shippey B, May A. The Scottish centre debrief model. International Journal of Healthcare Simulation. 2023.

[23] M Kolbe JW Rudolph 2018 What’s the headline on your mind right now? How reflection guides simulation-based faculty development in a master class BMJ Simulation and Technology Enhanced Learning 4 3 126 132 35520468 10.1136/bmjstel-2017-000247 PMC8990199

[24] J Brooks S McCluskey E Turley N King 2015 The utility of template analysis in qualitative psychology research Qual Res Psychol 12 2 202 222 27499705 10.1080/14780887.2014.955224 PMC4960514

[25] Symon G, Cassell C, King N. Doing template analysis. 2012. In: In Doing template analysis [Internet]. SAGE Publications, Inc.; [426–50].

[26] V Braun V Clarke 2006 Using thematic analysis in psychology Qual Res Psychol 3 2 77 101

[27] P Dieckmann S Molin Friis A Lippert D Ostergaard 2009 The art and science of debriefing in simulation: Ideal and practice Med Teach 31 7 e287 e294 19811136 10.1080/01421590902866218

[28] MM Dube J Reid A Kaba A Cheng W Eppich V Grant 2019 PEARLS for systems Integration: a modified PEARLS framework for debriefing systems-focused simulations Simul Healthc 14 5 333 342 31135684 10.1097/SIH.0000000000000381

[29] RM Fanning DM Gaba 2007 The role of debriefing in simulation-based learning Simul Healthc 2 2 115 125 19088616 10.1097/SIH.0b013e3180315539

[30] JW Rudolph R Simon RL Dufresne DB Raemer 2006 Thereʼs no such thing as “nonjudgmental” debriefing: a theory and method for debriefing with good judgment Simulation in Healthcare: The Journal of the Society for Simulation in Healthcare 1 1 49 55 10.1097/01266021-200600110-00006 19088574

