# Supplementary material for: ‘Everybody’s voice is important’: using translational simulation as a component of change management
Source: Adv Simul (Lond). 2025 Jul 5;10:38. doi: 10.1186/s41077-025-00364-0 (PMC12228184; doi:10.1186/s41077-025-00364-0)
Supplement: Supplementary file 1 — Supplementary Material 1 [file 41077_2025_364_MOESM1_ESM.pdf]

PESSI Simulation Programme, RHCYP
The experience of moving to a new site and the lived experience of change
Interview Questions and Post-It note reflection themes

Bartunek et al. / ON THE RECEIVING END 185

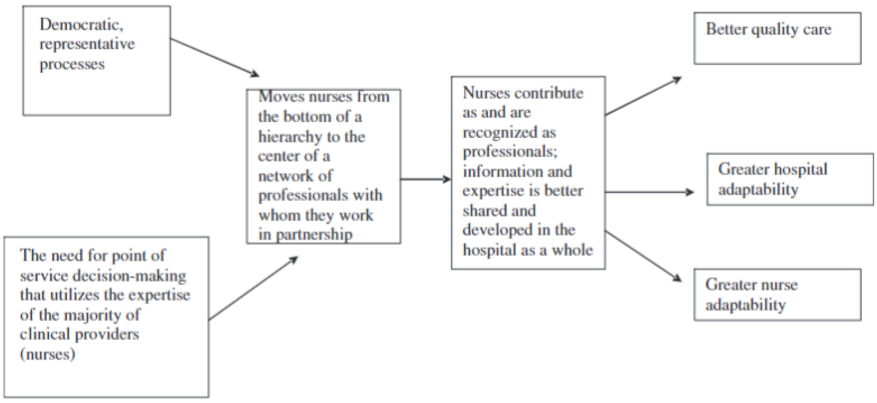

FIGURE 1: Schematic Summary of Porter-O'Grady Model of Shared Governance
SOURCE: Adapted from Porter-O'Grady and Finnigan (1984).

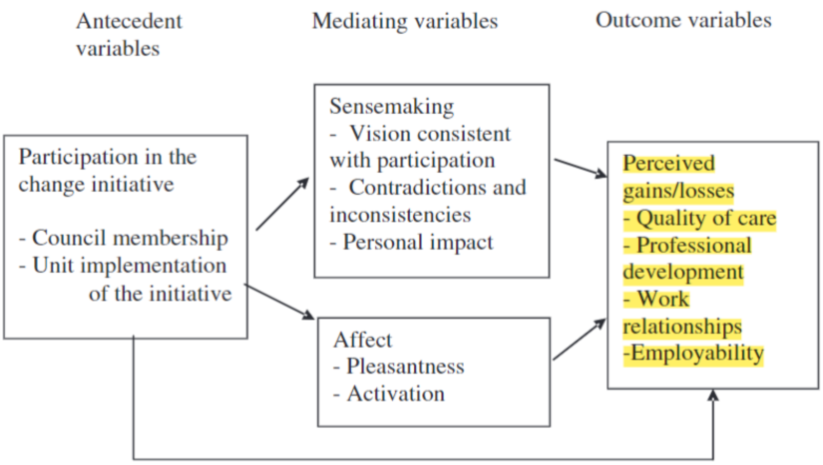

FIGURE 2: Conceptual Model: The Proposed Roles of Sensemaking and Affect on Assessments of Change

KEY THEMES FROM THE CONCEPTUAL FRAMEWORK:

1. Antecedent Principles
   a. Participation in the change
   b. Sense of partnership with ?whole team/?managment and 'say'
   c. Recognition of voice and (clinical/practical/on the ground) experience
2. Mediating variables
   a. Sense making of the change
      i. Vision "Do I see the big picture here and am I in it?"
      ii. ?Inconsistences? "Is the big picture consistent with my suggestions/input/vision?"
      iii. Personal impact – "Is this change good/bad for me?"
   b. Affect/emotion?
      i. Activation/?engagement "Do I see a future that is bright and that I want to be a part of creating?"
      ii. Ease of change – Vision "Do I see the big picture here and am I in it?"
      iii. Trust "Can I trust my manager/leader/organisation to live up to the promises of this change?" Will it be a good/stressful experience?
3. Outcomes
   a. Gains/Loses
   b. Care quality - Is care better?
   c. Team development - Is the team better?
   d. Individual development - Am I better?

POTENTIAL PRE-INTERVIEW QUESTIONS:

- How are you feeling about the move? Both positively and negatively?
    - Are you looking forward to it?
    - What are your anxieties about it? Any frustrations?
- What are the reasons you feel that way (both postive and negative)?/Why is that?
- Can you tell me about your sense of involvement in the move so far? How connected do you feel to it? How does that make you feel?
    - As a person? As part of a team?

POTENTIAL POST-IT NOTE REFLECTIONS ON THE DAY

1) (After the tour?) In just a sentence or two – can you give me some thoughts about how you are feeling right now about the day, and about the move?
2) (After the sim?) In just a sentence or two – can you give me some thoughts about how you are feeling right now about the day, and about the move?
3) (After the debrief?) In just a sentence or two – can you give me some thoughts about how you are feeling right now about the day, and about the move?

POTENTIAL POST INTERVIEW QUESTIONS (up to one month after moving)

- How are you feeling about the move now that you are here? Both positively and negatviely?
- Looking back, what have been the key moments/factors that have impacted your own experience of moving?
- How do you feel the SIM impacted on the move?
    - Did you notice any difference between yourself and colleagues who didn't take part in the simulation? If so can you expand on this?
- Can you tell me about your sense of involvement in the move?
- Thinking about the last few weeks, what do you think has been gained and/or lost because of the experience?
- Thinking about today:
    - How is patient care?
    - How is the team?
    - How are you as a professional and as a person?

Probing Qs

- Oh that's really interesting. Tell me more about that.
- Why do you think that is?
- What do you mean by that?
- Do you mind just expanding on what you mean there?
